# Diagnostic pitfalls: a case of prostate cancer and rectal cancer accompanied by prostate cancer invasion of the rectum

**DOI:** 10.1186/s13000-022-01282-9

**Published:** 2022-12-29

**Authors:** Qiongxian Long, Ji Wu, Yong Peng, Xuqian Zhang, Xinya Liu, Huaping Chen

**Affiliations:** 1 Department of Pathology, Nanchong Central Hospital, North Sichuan Medical University, Nanchong, China; 2 Department of Urological Surgery, Nanchong Central Hospital, North Sichuan Medical University, Nanchong, China; 3 Department of Gynaecology and Obstetrics, Nanchong Central Hospital, North Sichuan Medical University, Nanchong, China; 4 Department of Medical Iconography, Nanchong Central Hospital, North Sichuan Medical University, Nanchong, China

**Keywords:** Prostate Cancer, Rectal Cancer, Immunohistochemistry, Diagnostic Pitfalls

## Abstract

**Introduction:**

Case of double primary cancer of the prostate and rectum is rare, prostate cancer involving the postoperative intestinal anastomotic mucosal tissue is even rarer.

**Case presentation:**

We report a case of rectal cancer discovered 1 year after a diagnosis of prostate cancer and a tumour in the postoperative anastomotic intestinal mucosal tissue involving prostatic adenocarcinoma at 1 year after the diagnosis of rectal cancer. Due to the poor differentiation of both prostate and rectal cancers, there are some pitfalls in the diagnosis of intestinal mucosal lesions at an anastomosis. The lack of an accurate diagnosis of a tumour in anastomosis intestinal mucosal tissue will affect treatment and patient survival.

**Conclusions:**

The pathologists should have a detailed understanding of the patient's medical history and carefully observe the histopathological morphology and, if necessary, immunohistochemistry or other techniques should be used to assist in the pathological diagnosis and avoid both misdiagnosis and missed diagnosis.

## Introduction

Global cancer statistics from 2020 showed that the prostate and rectal cancer are among the top three cancers, in terms of morbidity and mortality, in men, prostate cancer is first and second in morbidity and mortality, whereas rectal cancer is third in morbidity and mortality [1]. Thus, these cancers seriously affect the men’s health. Denonvilliers’ fascia is a barrier that makes invasion of the rectum by prostate cancer a rare event. However, if it occurs, it can present with symptoms that mimic rectal cancer, such as constipation, abdominal pain, rectal bleeding, and altered bowel habits. Therefore, it is important to understand the disease. In this report, we describe a case of prostate cancer and rectal cancer accompanied by prostate cancer invasion of the rectum and discuss its clinical pathological features and diagnostic pitfalls.

## Case presentation

**Fig. 1:**
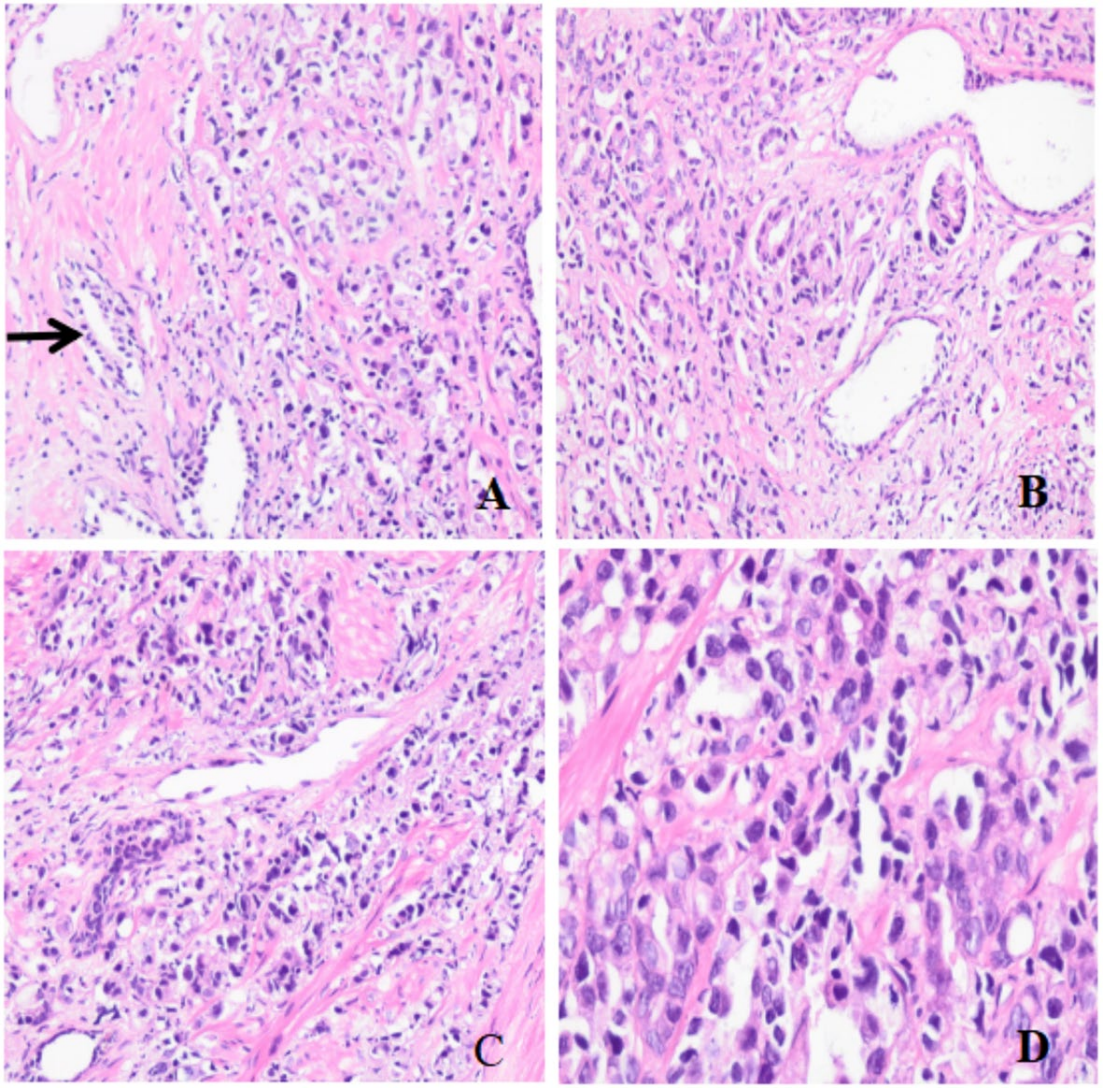
Adenocarcinoma of the prostate, Gleason score9 (5 + 4), H&E. **A** Benign prostate tissue (black arrow head, magnification ✕ 200). **B** Poorly formed glands (magnification ✕ 200). **C** Individual cells (magnification ✕ 200). **D** Atypical cells with prominent nucleoli (magnification ✕ 400)

In November 2017, a-70-year-old male patient was admitted with complaints of dysuria without bellyache and bloating for 6 months. Preoperative imaging examination showed no cancer in bone tissue. Laboratory studies showed a prostate-specific antigen (PSA) value of 47.43 ng/ml. In January 2018, the patient underwent radical resection of the prostate with pelvic lymph node dissection. Postoperative pathological examination of the tumour showed acinar adenocarcinoma with some intraductal carcinoma. The Gleason score was 5 + 4, and the International Society of Urological Pathology (ISUP) grade group was 5 (Fig. 1). Vessel carcinoma embolus, nerve invasion, and extraprostatic extension were observed. Tumour cells were found at the incisive margin of the bladder neck, other margins were negative. No cancer was detected in bilateral seminal vesicles. However, metastasis was detected in one of the pelvic lymph nodes. After surgery, androgen deprivation therapy (ADT) and radiotherapy (RT) were administered based on the treatment criteria and a diagnose of prostate cancer. Unfortunately, the patient voluntarily discontinued treatment in January 2019.

**Fig. 2:**
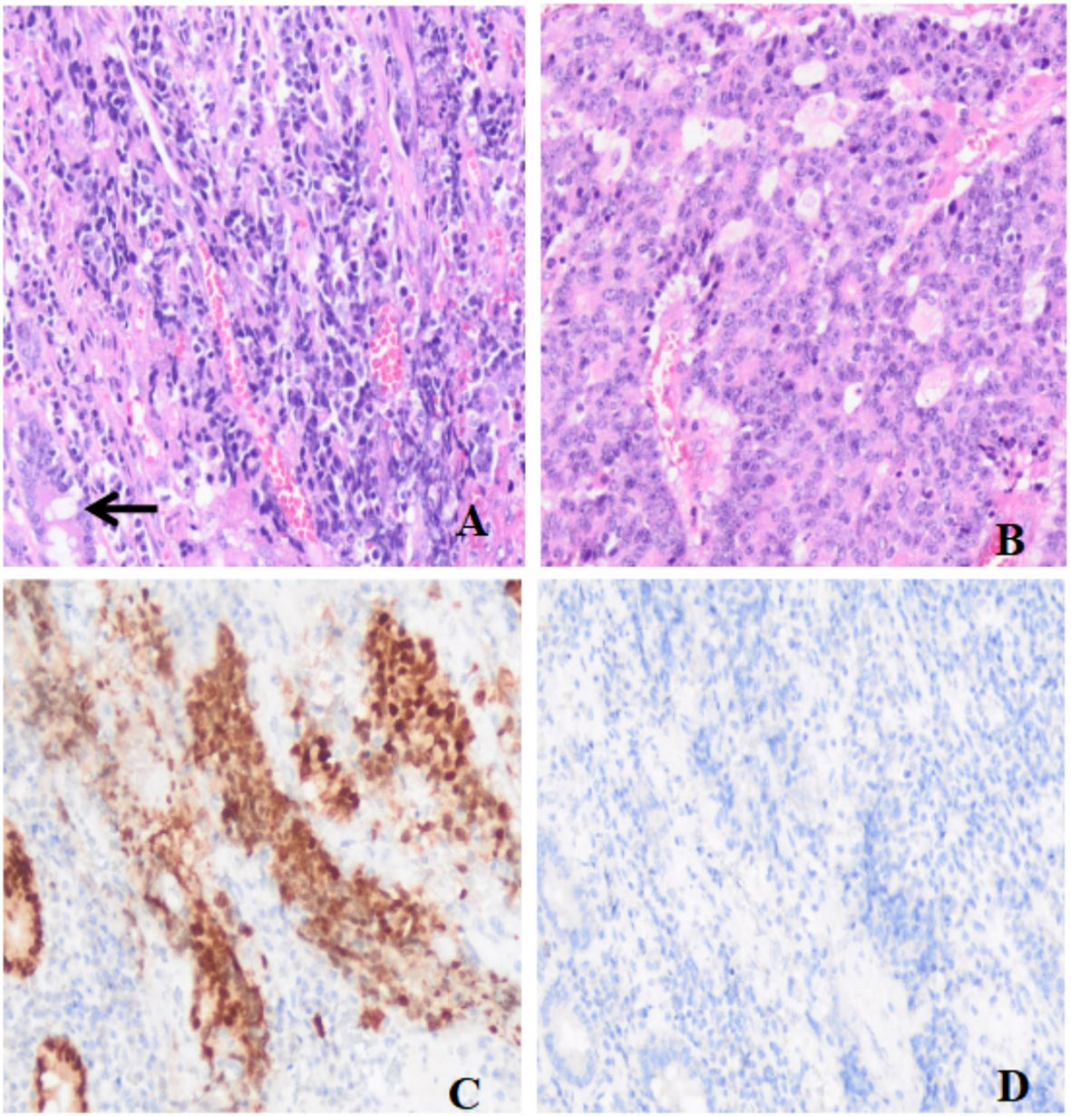
H&E and immunohistochemical (IHC) staining of the adenocarcinoma in the rectum (magnification ✕ 200). **A** Normal rectum mucosa epithelium (black arrow head). **B** Cribriform and fused glands. **C** Strong positive expression of CDX-2 in cancer and mucosal epithelial cells. **D** Negative for NKX3.1 in cancer and mucosal epithelial cells

In September 2019, the patient experienced mild abdominal distention, no abdominal pain, nausea, and vomiting. Electronic colonoscopy detected a lump in the rectum with circumferential growth, leading to a narrow lumen, and pathological examination revealed adenocarcinoma of the rectum. The patient underwent laparoscope-assisted radical resection of rectal carcinoma. Gross examination showed an ulcerated mass in the rectum wall measuring 4.5 × 3.5 x 2 cm. Microscopically, the tumour was a poorly differentiated adenocarcinoma of the rectum that infiltrated the entire wall of the intestine and formed nodules. No metastasis was detected in regional lymph nodes. Considering his history of prostate cancer, this case was re-examined by two senior pathologists, and the tissue type of the tumour was assessed by immunohistochemical staining. The case was eventually diagnosed as a primary adenocarcinoma of the rectum, ruling out the possibility of prostate cancer involving the rectum (Fig. 2). After surgery, the patient received adjuvant chemotherapy according to treatment standards and based on the diagnosis of rectal cancer and prostate cancer.

**Fig. 3:**
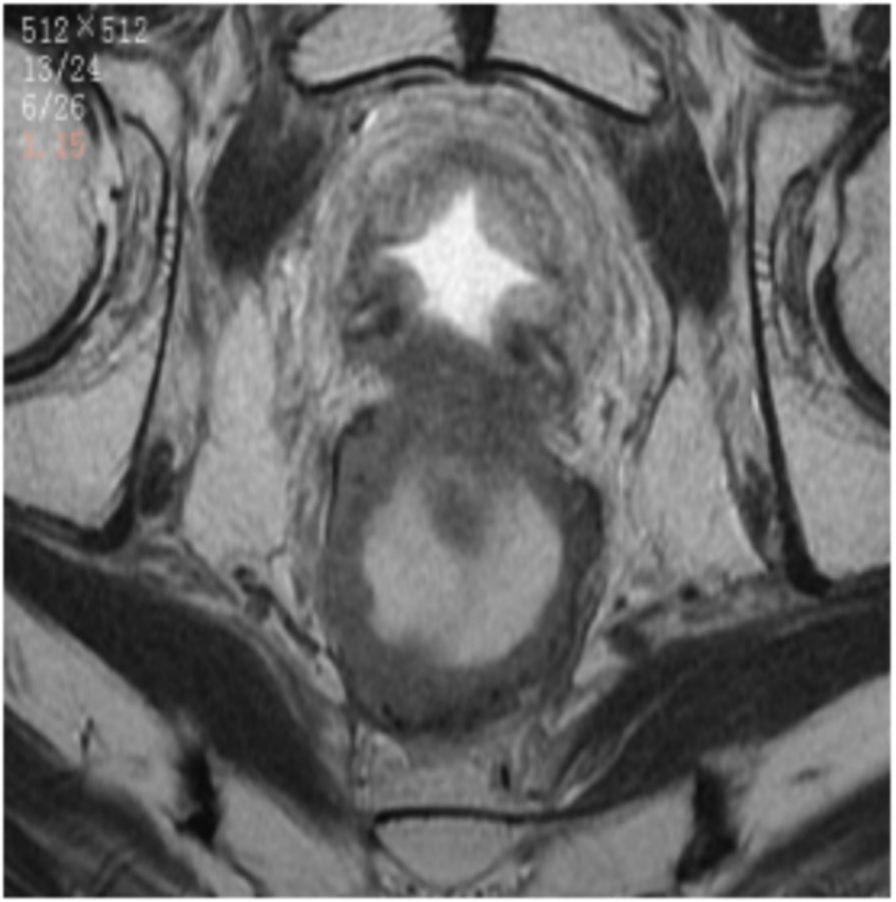
MRI showing irregular circumferential thickening of the rectum wall at the surgical site

**Fig. 4:**
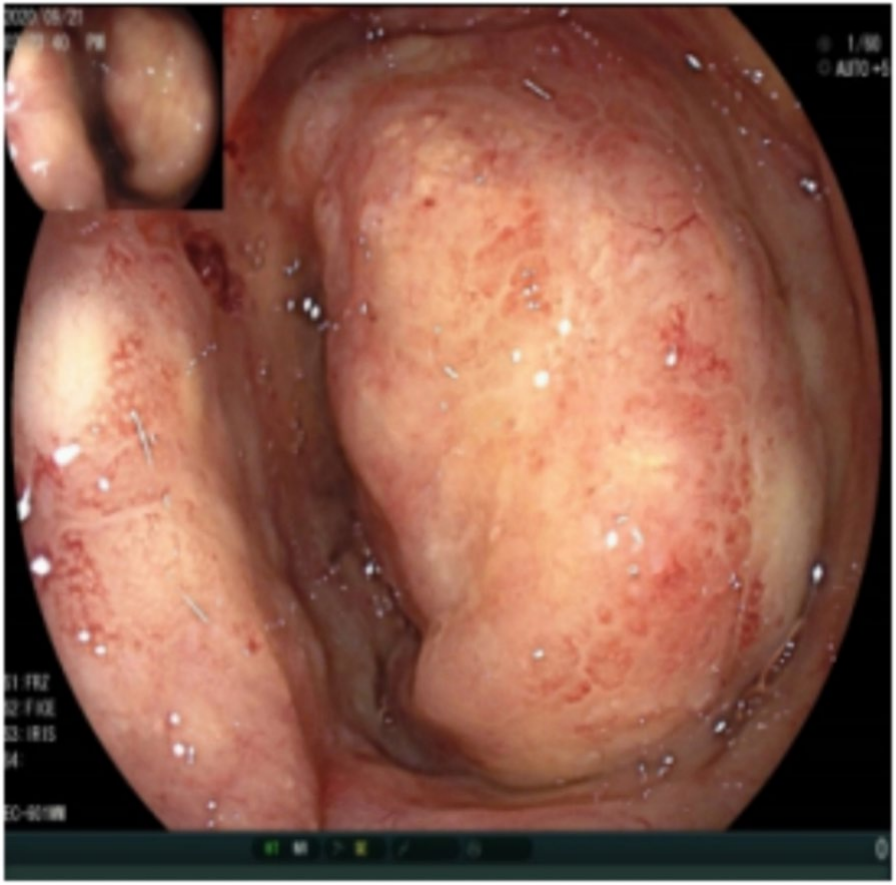
Colonoscopy showing a neoplasm in the intestinal lumen of the anastomosis

**Fig. 5:**
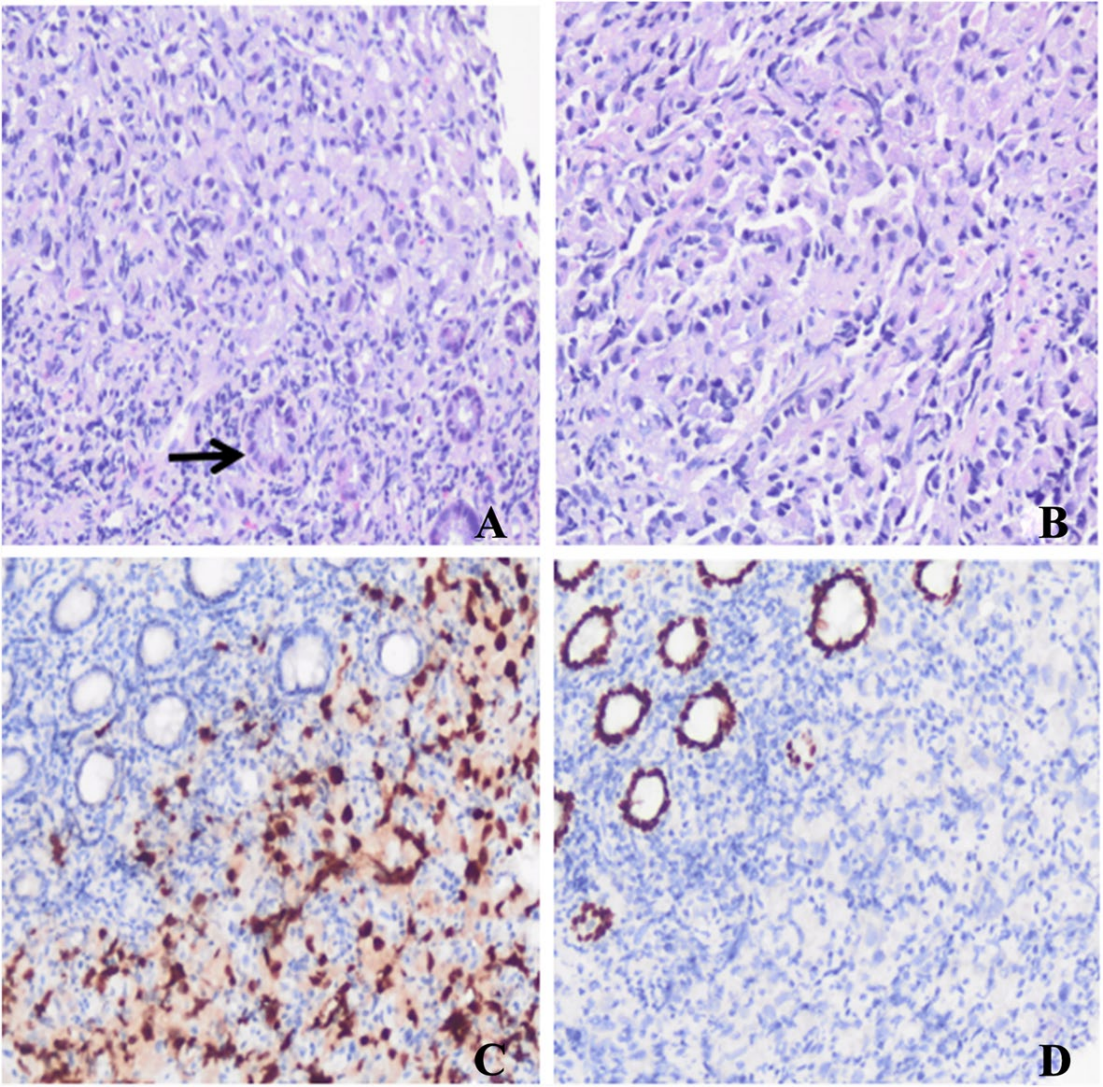
H&E and immunohistochemical (IHC) staining of the tumour at the anastomotic site (magnification ✕ 200). **A** and **B** H&E staining of the tumour, and normal rectum mucosa epithelium (black arrow head). **C** Strong positive expression of NKX3.1 in cancer cells. **D** Negative for CDX-2 in cancer cells

In September 2020, the patient went to the hospital for a follow-up examination. Laboratory studies showed a PSA value of 35.71 ng/mL, which is much higher than normal. MRI showed irregular circumferential thickening of the rectum wall and narrowing of the lumen at the surgical site (Fig. 3). To determine the reason for the residual rectum stricture, the patient underwent an electronic colonoscopy, which showed a neoplasm with a brittle texture and easy bleeding in the lumen of the anastomosis (Fig. 4). Microscopy showed diffuse epithelioid cells in the lamina propria of the intestinal mucosa at the anastomotic site. Because the cells were poorly differentiated, the tissue source was difficult to identify. Immunohistochemistry showed that the epithelioid cells within the lamina propria were positive for NKX3.1, PSMA, PSAP, and P504s, but negative for CK20, CDX-2, Villin, and SATB2 (Fig. 5). The pathologic diagnosis was prostate adenocarcinoma involving the intestinal mucosa at the anastomotic site.

At present, the patient is continuing endocrine therapy after RT, with regular outpatient follow-up, and is generally in good condition.

## Discussion

From an anatomical point view, the prostate is in the pelvis, under the urinary bladder and in front of the rectum. Prostate cancer and rectal cancer are the most common pelvic cancers in males [2]. Because of its location, prostate cancer often affects urination, ejaculation, and in some cases, defecation. It can invade nearby organs, including the rectum, bladder, and ureters, and can metastasize to the bones and lymph nodes [3–5]. There is a thick capsule between the prostate and rectal wall called Denonvilliers’ fascia [6]. Prostate cancer rarely invades the rectum. However, if it does, the patient can present with rectal-related symptoms, such as constipation, abdominal pain, rectal bleeding, and altered bowel habits [7]. Therefore, prostate cancer can be misdiagnosed as rectal cancer [8].

Curative treatment of prostate cancer mainly consists of prostatectomy with or without RT and/or ADT according to the estimated risk of recurrence [9]. Patients with prostate cancer who receive RT are at higher risk of secondary malignancies, such as bladder and rectal cancers [10–12]. In our case, rectal cancer was found 1.5 years after diagnosis of prostate cancer, which was treated with RT, therefore, it is likely that the rectal cancer was caused by RT for prostate cancer.

The patient initially presented with dysuria and was diagnosed with prostate cancer. A retrospective analysis of the patient’s medical records confirmed that the patient had no obvious intestinal symptoms at prostate cancer diagnosis, and MRI showed a normal intestinal wall. One year later, the patient developed intestinal symptoms. HE and immunohistochemical staining of tissue obtained by biopsy during colonoscopy led to a diagnosis of poorly differentiated adenocarcinoma. Therefore, the tumour in the rectum was a primary adenocarcinoma of the rectum, with no prostate cancer involvement. However, 1 year after surgery for the rectal cancer, MRI showed irregular circumferential thickening of the rectum wall and narrowing of the lumen at the surgical site. An intestinal examination was performed, and mucosal tissue was taken from the anastomotic site for pathological examination. Microscopy showed diffuse tumour cells in the lamina propria of the mucosa. The tissue origin was difficult to identify based on the HE histological morphology. This was the biggest challenge in diagnosing the case, because the intestinal mucosal tissue was taken from the anastomotic site, and the rectal tumour removed 1 year before consisted of poorly differentiated adenocarcinoma, a pathologist with limited experience may have diagnosed this as a recurrence of rectal cancer. Based on the pathological diagnosis of this case, we can make the following conclusions: (1) Before making the final diagnosis, the pathologist must study the patient’s history in detail. In this case, the pathologist knew that the patient had a history of prostate adenocarcinoma with poorly differentiated tumour components; therefore, it was important to determine whether the intestinal anastomotic lesions involved prostate adenocarcinoma. (2) Pathologists should carefully combine all the previous pathological examination results and comprehensively analyse all possibilities for the current lesion. In this case, pathological examination after the first surgery for prostate cancer showed residual cancer at the margin of the bladder neck, indicating that the prostate lesion had not been completely removed. The bottom of the bladder is adjacent to the rectum, and if cancer is present on the edge of the bladder neck, it is likely to involve the rectal wall as the disease progresses. If pathologists analyse these findings, even if the tissue source of the tumour in the anastomotic tissue cannot be determined based on the histological morphology of HE-stained tissue, it will not be directly diagnosed as a recurrence of rectal cancer. (3) Pathologists should carefully observe the HE morphology of the tissue and look for all clues related to the diagnosis. (4) For poorly differentiated tumours with an uncertain tissue origin based on histological morphology, appropriate immunohistochemical markers can be used to assist diagnosis. Traditionally used biomarkers to confirm carcinoma of prostatic origin are PSA and prostatic acid phosphatase (PAP) [13]. However, these markers are often only focally expressed in metastatic prostatic carcinoma, with reported detection rates of ~ 80% for PSA and ~ 60% for PAP [14, 15]. Previous studies demonstrated that NK3 homeobox 1 (NKX3.1) is generally very specific to prostatic origin, and the sensitivity of NKX3.1 was 90.9% -100% [14, 16]. For colorectal cancer, SATB2 was a relatively specific immunohistochemical marker for determining the origin of adenocarcinomas and distinguishing primary ovarian mucinous adenocarcinomas from colorectal metastases [17]. In this case, we used immunohistochemistry to detect PSAP, NKX3.1, CK20, and SATB2 in the intestinal mucosal at the anastomotic site. The results showed that the lesions in the anastomotic mucosa involved prostatic adenocarcinoma rather than a recurrence of rectum adenocarcinoma.

After discovering prostatic adenocarcinoma in the intestinal mucosa of the anastomotic site, the oncologist adjusted the treatment according to the progression of the tumour, which prolonged survival of the patient. At present, the patient’s condition is stable. Misdiagnosis of the lesion in the postoperative intestinal anastomotic mucosal tissue as a recurrence of rectal adenocarcinoma, would ignore the progression of prostatic adenocarcinoma, which would delay treatment, further accelerate the progression of prostatic adenocarcinoma, and shorten the patient’s survival. Therefore, pathologists should have a detailed understanding of the patient's medical history, carefully observe the histopathological morphology, and if necessary, use immunohistochemistry or other techniques to assist the pathological diagnosis and avoid both misdiagnosis and missed diagnosis.

## Data Availability

All data generated or analyzed during this case are included within the article.
